# Role of persistent CMV infection in configuring T cell immunity in the elderly

**DOI:** 10.1186/1742-4933-4-2

**Published:** 2007-03-21

**Authors:** Sonya Vasto, Giuseppina Colonna-Romano, Anis Larbi, Anders Wikby, Calogero Caruso, Graham Pawelec

**Affiliations:** 1 Gruppo di Studio sull'Immunosenescenza, Dipartimento di Biopatologia e Metedologie Biomediche, University of Palermo, Italy; 2 University of Tübingen Medical School, Center for Medical Research, ZMF, Tübingen, Germany; 3 Department of Natural Science and Biomedicine, School of Health Sciences, Jönköping University, Box 1026, 551 11 Jönköping, Sweden

## Abstract

Ageing is associated with declines in many physiological parameters, including multiple immune system functions. The rate of acceleration of the frequency of death due to cardiovascular disease or cancer seems to increase with age from middle age up to around 80 years, plateauing thereafter. Mortality due to infectious disease, however, does not plateau, but continues to accelerate indefinitely. The elderly commonly possess oligoclonal expansions of T cells, especially of CD8 cells, which, surprisingly, are often associated with cytomegalovirus (CMV) seropositivity. This in turn is associated with many of the same phenotypic and functional alterations to T cell immunity that have been suggested as biomarkers of immune system aging. Thus, the manner in which CMV and the host immune system interact is critical in determining the "age" of specific immunity. We may therefore consider immunosenescence in some respects as an infectious state. This implies that interventions aimed at the pathogen may improve the organ system affected. Hence, CMV-directed anti-virals or vaccination may have beneficial effects on immunity in later life.

## Background

Ageing is associated with multiple immune system dysfunctions [1]. An important current direction for immunosenescence research is towards assessing the clinical impact of age-associated modifications of immunity and modulating them. Infectious disease is increased in frequency and severity in the elderly, implying that age-associated decreasing immunity results in increased incidence in those diseases which the immune system has evolved to combat. It is well-established that the incidence and case fatality-rate of infections like tuberculosis, pneumonia, bacteraemia and cholecystitis are increased in elderly people [2]. In a study of Japanese women, the annual death rate from pneumonia continued to accelerate with age, whereas stroke, lung and stomach cancer rates plateaued [3].

Increased infections in older people can be explained by several interacting factors. First, decreased immune function may be due to hidden diseases and/or medication and/or malnutrition. Ageing *per se *is associated to loss of integrity of physical barriers along with decreased hygiene that lead to an increased susceptibility to infection. Secondly, to decreased immunocompetence towards certain pathogens. Finally, iatrogenic causes (eg. urinary catheter) and housing conditions (eg. nursing houses, geriatric wards and houses for the elderly) may also contribute to increased infections in the elderly particularly due to nosocomial infection [2].

Senescence of the clonotypic immune system is thought to be principally the result of a non-adapted T cell response to stimulation. Many age-associated changes in T cell phenotype and function are caused by the process of senescence [4]. Lifelong and chronic antigenic load may represent the major driving force for immunosenescence, which impacts on human lifespan by reducing the number of virgin antigen-non experienced T cells, and results in their replacement by expanded clones of antigen-experienced effector and memory T cells which display a late differentiation phenotype. Gradually, the T cell population shifts to a lower ratio of naïve cells to memory cells, the thymus releases fewer naïve T cells with age and those T cells remaining, especially the CD8+ subset, also show increased oligoclonality with age. Thus, the repertoire of cells available to respond to antigenic challenge from previously unencountered pathogens shrinks. In addition, older organisms often are overrun by memory cells that carry a single type of T cell receptor, i.e. the clonal expansion referred to above. Thus, the memory cells from old individuals might recognize a limited set of antigens despite being plentiful in number, and in addition, are likely to show various degrees of dysfunctionality. Many of the clonal expansions filling the individual's immune system seem to result from previous infections by persistent viruses, especially CMV, but also, to a lesser extent, EBV and possibly other herpesviruses. A high number of CD8+ cells are found to be specific for a single epitope of cytomegalovirus: in some individuals up to a quarter of circulating CD8 cells carry receptors for a single CMV epitope [5-7]. T cells subsets commonly carry the CD28 surface molecule, which co-stimulates the cells to divide when an antigen is presented by the APC. However, old memory cells, i.e. CD8 cells that have encountered the antigen a long time ago and have been chronically stimulated over time, tend to lose CD28 and, as a result, multiply less robustly when exposed to antigens than do younger cells (Table 1). The hypothesis derived from the above findings is that T cell immunosenescence and, possibly, mortality and morbidity will occur earlier in people that have been exposed to an antigenic overload (due to chronic infections). The opposite will apply both in people exposed to a lower antigenic load and in people equipped with an immunogenetic background able to more efficiently deal with these types of infections. This means that the ability of our immune system is progressively worn out by the requirement for constant immunosurveillance against persistent antigen sources. In developed countries, improved hygienic conditions and the consequently lower level of bacterial contamination of food and water could have reduced the antigenic overload preserving the immune system from rapid exhaustion and helping a large number of individuals to extend their longevity [4,8-11].

**Table 1:** Alterations in T cell compartments with age

T cell
↑ CD45RO^+ ^cells
↑ CD95^+ ^cells
↓ CD28 expression
↑ CD152 expression
↑ inhibitory receptors expression
↓ signal transduction (rafts)
↓ cytokine production (IL-2)
↓ apoptosis of CD8 cells
↑ apoptosis of CD4 cells

## Octo/nona longitudinal studies

The use of a longitudinal design in ageing research is highly desirable for conducting population-based studies by advantages in identifying age-related alterations and the detection of intra-individual changes [12]. Although the longitudinal design is a superior alternative to the cross-sectional method for conducting ageing research it has seldom been employed because of extensive costs, need of careful coordination and long duration. Particularly rare are longitudinal studies on individuals over 85 years of age, the group of people deliberately focused on in the Swedish OCTO/NONA longitudinal studies [6]. These studies initially examined age-related changes in a limited number of immune parameters but are now performing more sophisticated immunological assessments. There are some parameters which are important for successful ageing, which can only be revealed by such longitudinal studies. At first, a cluster of immunological parameters which define an immunological risk phenotype predicting 2-year mortality in very old individuals was established in the earlier OCTO study [13]. The term "Immunological Risk Profile" (IRP), took into consideration elements like an inverted CD4:8 ratio, poor T cell proliferative responses to mitogens, low interleukin-2 production, increased numbers of CD8-positive CD28-negative cells, decreased B cell count, and later, CMV seropositivity, which also seems to play an important role in determining the IRP (Table 2) [6,7].

**Table 2:** Characteristics of the immune risk phenotype

CD4:8 ratio < 1
poor T cell proliferative responses to mitogens
increased CD8+CD28-negative and CD8+CD57+ cells
low B cells count
CMV-seropositivity
clonal expansions of CD8 cells carrying receptors for CMV
high proportion of dysfunctional cells amongst the CMV-specific CD8 cells

## CMV

Human CMV is a persistent herpes virus that is present in approximately 50% of the adult population and may reach 90% in the elderly in Europe. CMV infection in immunocompetent individuals is normally asymptomatic, but can be a major cause of morbidity in immunosuppressed individuals and susceptible neonates. The control of CMV infection by the immune system is then of major importance to delay/decrease its effect on the individual's health [14]. There are reports where it appears that CMV infection could alter the composition of the different T cell subsets, showing an increased number of CD8+CD57+ subset in CMV-seropositive individuals. In earlier studies, it has been reported that CMV seropositivity is associated with an increased number of both CD4+ and CD8+ CD28-negative cells. Independently, this phenotype has also been associated with age. However it may in fact be primarily associated with CMV status and only secondarily with age, linking the increased frequency of CMV infection to age. But both age and CMV status influence the number of CD8+ cells and the expression of CD45RA and CD28 [14-17].

One of the immunodominant viral antigens recognized by CMV specific CD8+ T cells, at least in HLA-A2+ donors, is derived from the 65-KDa phosphoprotein (pp65). From this protein the immunodominant epitope presented by HLA-A*0201 has been used to investigate the frequency of CD8+ cells against CMV. The OCTO and NONA samples have been studied using MHC/peptide tetramers and preliminary data revealed that a large number of CD8 and CD28-negative cells where specific for the pp65 antigen. It has become evident that a subset of CD8+ cells, generally of the memory phenotype, expresses inhibitory NK receptors which can block CTL effector function. Together with the well-known phenomenon of CD8 clonal expansions presented in the elderly, these findings imply a co-dominant role of CMV as a cause for a compromised immunity in old age. Mainly, two different approaches have tried to pinpoint the influence of CMV on the ageing of the human immune system. The first was the development of tetramer technology to directly identify T cells carrying receptors for single peptide epitopes; and the second, the development of longitudinal studies of naturally ageing populations. Importantly, this study also indicated that the risk phenotype was independent of the individuals' health status at that particular time. [4-6,18-20].

## CD8

The clonal expansion of CMV-specific CD8+ T cells which accumulate progressively with age resemble CD8+ T cells that are driven to the end-stage of replicative senescence in cell culture in response to repeated rounds of antigen-driven proliferation, and which may also be found in cancer patients (21). Senescent CD8+ T cell cultures show downregulation of CD28 expression and shortened telomeres. In fact, the oligoclonally expanded cells within the CD8+CD28- T cell population generally have shorter telomeres than the CD28- subset as a whole, suggesting extensive proliferation, presumably in response to chronic antigenic exposure [22]. The majority of the CD8+ T cells specific for CMV in the elderly is probably dysfunctional at least in terms of IFN-γ production (but this may also be a property of end-stage differentiation effector CTL). A minority of CMV-specific CD45RA+ reverted cells found in the elderly, however, constitute a different population: one which remains capable of proliferation and interferon secretion, like the majority of CD28- cells with the same CMV specificity in young donors. Antigen-specific dysfunctional T cells have been also described in EBV, HIV and HCV infected patients, melanoma and renal carcinoma patients and in rheumatoid arthritis patients. In all these conditions, chronic antigenic stress may result in the accumulation of clonal expansions of dysfunctional "anergic" T cells partly due to apoptosis resistance. This is still matter of debate but this mechanism might contribute to the increased CD8+ antigen-specific T cell number under conditions of chronic antigen stresses. This could also be true in the young population subjected to persistent antigenic stresses, particularly cancer patients in developed nations and parasite-infected patients in less-developed nations. This supports the concept of immune exhaustion due to pathogen or tumor escape from the immune system [21-25].

## CD28

CD28 is an important receptor affecting both T cell activation and susceptibility to apoptosis, the expression of which is well-documented as being decreased in aged individuals. CD28 costimulation is required for T cell activation and to prevent anergy and to activate telomerase. Together with telomere shortening, loss of CD28, especially together with loss of CD27, can be taken as a marker for T cell replicative senescence and/or end-stage differentiation [22]. Both the OCTO/NONA studies showed a significant decrease of CD28 expression by CD8 cells of old compared to middle-aged individuals. This was particularly marked in old individuals with an inverted CD4/8 ratio of <1, who are most at risk for incipient mortality [25]. Stimulation of T cells via T cell receptor and CD28 leads to the activation of a myriad of signalling pathways that ultimately induce the translocation of transcription factors such as NF-AT and NF-kB to the nucleus initiating IL-2 production, or other effector functions.

Clonal expansion and effector function are then possible. Most investigators agree that the CD8+CD28- population increases with age and CMV seropositivity. Nevertheless, these cells are still able to respond to certain stimuli such as IL-15 which preferentially extends CD8+ memory cell lifespan. The recent discovery of membrane rafts may help to understand why cells respond to some stimuli and not to others. Membrane rafts are specialized membrane microdomains where the signalling platform is formed. Surprisingly, CD8+ cells possess a pre-assembled signalosome that might account for their ability to maintain CD28 signalling (via Akt phosphorylation) despite CD28 downregulation to apparently minimal or zero levels [26]. The use of *in vitro *aged T cell clones (TCC) allows accurate discrimination between CD28-low and CD28-negative cells. In vivo, it may also be the case that CMV-specific CD8+ expanded T cells still retain some CD28 on the surface. Our preliminary data using TCC derived from young, OCTO and centenarian donors show that Akt activation was inducible although these cells were classified in regular flow cytometry as CD28-negative. The maintenance of CD28 signalling can be attributed to the lateral mobility of membrane rafts which can concentrate the remaining CD28 molecules to the site of stimulation. Expression levels of CD28 can still be taken as a marker of immune senescence and cell replication. However, CD28 downregulation alone is not a marker for loss of functions since CD28 localization is as important as its expression level [27]. Membrane rafts are not only crucial for T cell activation but also for other processes such as apoptosis commitment. The Fas-dependent pathway was shown to involve death-inducing signalling complex (DISC) formation in membrane rafts [28]. Since changes in membrane raft properties have been found in CD4+ T cells from elderly individuals there is the possibility that CD8+ rafts also undergo changes due to repeated rounds of stimulation. These changes can lead to a differential capacity of DISC formation and could account for the resistance to apoptosis thought to play a role in the accumulation of CMV-specific CD8+ cells.

## KIR

Antiviral CD8+ T cell responses are regulated by killer cell inhibitory receptors, which can be divided into two structural types, killer immunoglobulin-like receptors (KIR) and killer cell lectin-like receptor (KLR) belonging to the C-type lectin family. The inhibitory receptor KLRG1 is expressed on 50% of CD8+ cells in young donors, whereas in the elderly the percentage rises to 80%. A distinguishing characteristic of the majority of KLRG1+ cells is that they are unable to undergo further clonal expansion. Furthermore, about 90% of CMV tetramer-positive CD8+ T cells in the elderly are also positive for KLRG1 compared to 70% in the young. Thus, persistent infection with CMV may lead to a gradual increase in the already high number of KLRG1+CD8+ cells during ageing. In the absence of TCR engagement, expression of KIR on non-self-reactive CD8+ cells is lowered to levels that no longer inhibit T cell function, suggesting that KIR expression is sustained by continuous encounter with antigens [29]. It also been seen, using intracellular cytokine staining, that the proportion of cells producing IFN-γ after specific antigen stimulation is higher in KLRG1+ CD8 cells regardless of age. However, CD8+ cells from old donors seem to produce less IFN-γ than young donors, regardless of KLRG1 expression. Nevertheless, CD8+KLRG-1+ cells are significantly more resistant to apoptosis. Recently, it was shown that the KIR2DL2 receptor was associated to membrane rafts and inhibited immune synapse formation and cellular activation in CD4+ T cells [21,23-25,29]. Further studies in CD8+ T cells are needed to test the hypothesis that inhibitory receptors are involved in CD8+ T cell anergy.

## Accumulation of dysfunctional cells

In addition to the persistent antigenic stimulus of the latent virus, age-associated dysregulation of apoptosis could account for the accumulation of dysfunctional CD8+ cells. In contrast, CD4+ cells seem to become increasingly susceptible to apoptosis with age, at least in vitro [20]. Increased resistance of CD8+ cells and increased susceptibility of CD4+ cells to apoptosis could help to explain the inverted CD4:8 ratio in the elderly, part of the IRP. In addition, telomerase activity, which compensates for the telomere attrition associated with the massive clonal expansion of virus-specific CD8+T cells in the blood and also in the lymphoid tissue, is progressively diminished in CD8+ T cells during repeated rounds of antigen-driven proliferation [30]. In IRP elderly, dysfunctional CMV-specific CD8+ T cells may accumulate because of compromised apoptotic pathways. Homeostasis is thought to maintain a constant number of T cells in the periphery by T cell proliferation, accumulation, death or renewal. Although thymic output is maintained in elderly individuals, at least to some degree, the difficulty in identifying naïve cells in the very elderly suggests that this may contribute to increased susceptibility to infectious disease. Hence, as most of the old individuals are CMV-positive, together with thymic involution, this might result in a marked shrinkage of the T cell repertoire for novel antigens. These data are consistent with the hypothesis that in the elderly the "immunological space" is occupied by dysfunctional anergic clonally expanded T cells specific for very few epitopes. This situation would leave the individual likely to be more prone to infectious disease and possibly also cancer, and may contribute to explaining the greater incidence of those diseases in elderly [5-7,9,25,31,32]

## Future perspectives

There remain many uncertainties still to clarify such as: a) Is there an immunogenetic component influencing the IRP phenotype that might explain the different degree of CMV clonal expansion vs non-IRP phenotype? b) May this difference depend on social or/and environmental factors? c) Might the genetic or environmental component affect the degree of clonal expansion of CMV in IRP individuals? d) What can be the main cause of death in IRP group vs non-IRP? e) Can IRP selection be predictive in young as well as in old individuals? f) Is it possible to revert/prevent accumulation of CMV-specific cells?

Human studies will require knowledge at least of the immunological history of the individual to be able to answer the above questions. Unfortunately, this is logistically and financially problematic, but it is to be hoped that Europe-wide studies may be established in he near future to explore these issues. The answers to all these questions will have practical implications and provide a route to novel interventions to understand and improve overall health and well-being in old age.
